# Preparation and evaluation of cyclodextrin polypseudorotaxane with PEGylated liposome as a sustained release drug carrier

**DOI:** 10.3762/bjoc.10.292

**Published:** 2014-11-25

**Authors:** Kayoko Hayashida, Taishi Higashi, Daichi Kono, Keiichi Motoyama, Koki Wada, Hidetoshi Arima

**Affiliations:** 1 Graduate School of Pharmaceutical Sciences, Kumamoto University, 5-1 Oe-honmachi, Chuo-ku, Kumamoto 862-0973, Japan; 2 Nihon Shokuhin Kako Co., Ltd., 30 Tajima, Fuji, Shizuoka 417-8539, Japan; 3 Program for Leading Graduate Schools “HIGO (Health life science: Interdisciplinary and Glocal Oriented) Program”, Kumamoto University, 5-1 Oe-honmachi, Chuo-ku, Kumamoto 862-0973, Japan

**Keywords:** cyclodextrins, doxorubicin, PEGylated liposome, polypseudorotaxane, sustained release

## Abstract

Cyclodextrins (CDs) can form polypseudorotaxanes (PPRXs) with drugs or drug carriers possessing linear polymers such as polyethylene glycol (PEG). On the other hand, PEGylated liposomes have been utilized as a representative anticancer drug carrier. However, little is known about the formation of CD PPRX with PEGylated liposome. In the present study, we first report the formation of CD PPRX with PEGylated liposome and evaluate it as a sustained release drug carrier. PEGylated liposome encapsulating doxorubicin was disrupted by the addition of α-CD. Meanwhile, γ-CD included two PEG chains and/or one bending PEG chain of PEGylated liposome and formed PPRX without the disruption of the membrane integrity of the PEGylated liposome. Moreover, the release of doxorubicin and/or PEGylated liposome encapsulating doxorubicin from the PPRX was prolonged in accordance with the matrix type release mechanism. These findings suggest the potential of γ-CD PPRX as sustained release carriers for PEGylated liposome products.

## Introduction

Cyclodextrins (CDs) are cyclic oligosaccharides comprising six (α-CD), seven (β-CD), and eight (γ-CD) glucopyranose units. They are characterized by a hydrophobic central cavity and a hydrophilic outer surface [1–2]. CDs are acknowledged to form inclusion complexes with various hydrophobic drugs, and improve their pharmaceutical properties [3]. For instance, γ-CD forms inclusion complexes with doxorubicin (DOX) with a stability constant of 345 M^−1^ [4]. CDs interact with cholesterol, phospholipids and proteins of biological membranes in the higher concentration range. Thus, CDs are utilized for studying the functions of caveolae, lipid rafts, and cholesterol transporters in various fields of cell biology [5]. Interestingly, CDs can also form inclusion complexes with linear polymers. Harada et al. have reported that a number of α-CDs spontaneously thread onto polyethylene glycol (PEG) and form necklace-like supramolecular assemblies [6–7]. The latter are referred to as polypseudorotaxanes (PPRXs), since the release of α-CD from the polymer chain can be achieved upon dissolution in water. The assembly of PPRX complexes is a size-dependent process, whereby the small cavity of α-CD assembles with PEG, while the large cavity of β-CD forms the PPRX with polypropylene glycol (PPG) [6–8]. In addition, γ-CD forms PPRX with double strand PEG chains [9]. In this case, γ-CD not only includes two extended PEG chains but also one bent PEG chain. Actually, our research group and Gao et al. have reported that γ-CD can form PPRXs with bulky molecules-appended PEG derivatives, implying the formation of γ-CD PPRX with one bent PEG chain [10–11]. On the other hand, the covalent capping of both ends of the polymer chains in PPRXs with bulky molecules results in the trapping of CDs, which in this case cannot be de-threaded from the assembly, hence giving rise to polyrotaxanes [12–13].

Recently, PPRXs and polyrotaxanes have been utilized as drug carriers for low-molecular weight drugs [14–15], protein drugs [16–17], and nucleic acids [18–20]. We have also developed a number of PPRXs with various drugs or drug carriers and utilized them as controlled release systems. For example, γ-CD formed PPRX with coenzyme Q10, improving the solubility and bioavailability of coenzyme Q10 [21–22]. Also, α- and γ-CDs formed PPRXs with PEGylated proteins and provided sustained release profiles of PEGylated insulin and PEGylated lysozyme in vitro and in vivo [10,23–25]. Furthermore, α- and γ-CDs PPRXs with PEGylated PAMAM dendrimer and α-CD-appended PEGylated PAMAM dendrimer were useful as sustained gene transfer carriers [26–27].

Liposomes (LPs) are microscopic phospholipid vesicles with a bilayered membrane structure and are used as a promising drug carrier [28]. When conventional LPs are administrated intravenously, they are coated with plasma proteins, which results in a rapid removal from the systemic circulation by the reticuloendothelial system (RES). To produce long-circulating LPs, hydrophilic polymers, carbohydrates, peptides and proteins have been used to modify the surface of LPs [29]. Additionally, the targeting efficiency of LPs has been improved by appending various targeting-ligands such as antibodies, sugars and folic acid to LPs [30–32]. Recently, stimulus responsive LPs such as bubble LPs, pH responsive LPs and thermoresponsive LPs have been developed as smart drug carriers [33–35].

PEGylated LP (PEG-LP) is one of the most popular LP products and forms a hydrophilic layer on the surface of LPs [28]. It is not recognized by RES, which leads to a prolonged retention in circulation (stealth characteristics), and shows the enhanced permeability retention (EPR) effect [36]. PEG-LP is widely used as a drug carrier to realize the targeted drug delivery of anticancer drugs [28]. PEG-LP encapsulating DOX is commercially available as DOXIL/CAELYX^®^ [28]. In addition, PEG-LPs are also utilized as long-circulating drug carriers for protein drugs and nucleic acids [37–38]. Thus, PEG-LPs are representative drug carriers and CD PPRXs of PEG-LPs could be promising long-acting drug carriers. However, little is known about the formation of PPRXs with CDs.

CDs are known to disrupt LP due to their interaction with membrane components such as phospholipids and/or cholesterol at higher concentration [39–40], even though CD PPRXs are generally prepared with CD solutions at high concentration. Therefore, it should be taken particular care to prepare CD PPRXs of PEG-LP. In the present study, we report on the first preparation and evaluation of CD PPRXs with PEG-LP as a sustained release drug carrier.

## Results and Discussion

### Interaction of phospholipid with CDs

**Figure 1:**
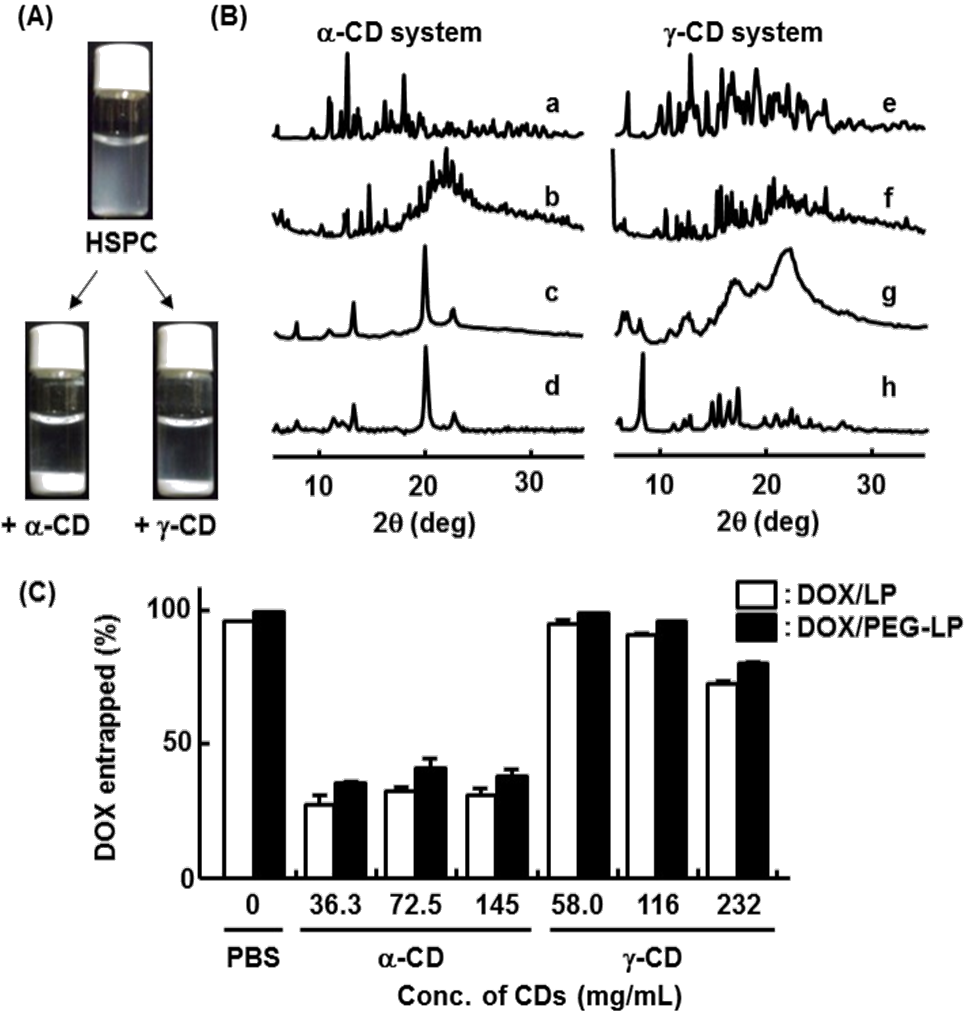
(A) Photographs and (B) powder X-ray diffraction patterns of precipitates formed by mixing the CD solutions and HSPC suspensions. (a) α-CD alone, (b) HSPC/α-CD physical mixture, (c) HSPC/α-CD complex, (d) PEG/α-CD PPRX, (e) γ-CD alone, (f) HSPC/γ-CD physical mixture, (g) HSPC/γ-CD complex, (h) PEG/γ-CD PPRX. (C) Effects of CDs on the entrapment ratio of DOX into LP or PEG-LP.

CDs disrupt LP by their interaction with lipid membranes. In particular, α-CD strongly interacts with phospholipids [5,39–40]. On the other hand, to prepare CD PPRXs, PEG should react with CDs in aqueous solution at high concentration of CDs. Thus, we should take particular care to prepare CD PPRXs of PEG-LP. First, to examine the interaction between CDs and phospholipids, both compounds were mixed in phosphate-buffered saline (PBS). As shown in Figure 1A, α- and γ-CDs formed precipitates with hydrogenated soybean phosphatidylcholine (HSPC) after mixing in PBS. Then, the powder X-ray diffraction was measured after collecting the precipitates (Figure 1B). Both precipitates showed the channel-type crystal patterns [41] which indicate the interaction of α- and γ-CDs with HSPC.

Next, to investigate disruptive effects of CDs on LP encapsulating DOX (DOX/LP) or PEG-LP encapsulating DOX (DOX/PEG-LP), DOX retained into DOX/LP or DOX/PEG-LP was quantitated 12 h after incubation with CDs (Figure 1C). After the incubation with 36.3–145 mg/mL α-CD solutions, approximately 70% and 60% of DOX was released from DOX/LP and DOX/PEG-LP, respectively, although the concentration dependence of the DOX entrapment ratio was not observed in the α-CD system. These concentrations of α-CD are high enough to completely disrupt the liposome membrane [42], which implies that the DOX entrapment ratio reaches the plateau at 36.3 mg/mL α-CD. In addition, the DOX entrapment ratio in the α-CD systems may be somewhat high. Conceivably, some DOXs may form aggregates in this experimental condition. These results, however, suggest the disruptive effects of α-CDs on DOX/LP and DOX/PEG-LP, although additional evaluations are required. Meanwhile, in the case of the γ-CD system, more than 90% of DOX was retained into DOX/LP or DOX/PEG-LP after the incubation at 58 or 116 mg/mL γ-CD, although more than 20% of DOX was released from DOX/LP or DOX/PEG-LP in the presence of 232 mg/mL γ-CD. These results suggest that DOX/LP and DOX/PEG-LP are not disrupted by 58 or 116 mg/mL γ-CD, even though 36.3–145 mg/mL α-CD and 232 mg/mL γ-CD show disruption effects on DOX/LP and DOX/PEG-LP. Hereafter, a fluorescence quenching experiment should be performed to obtain the additional evidences that LP structure is kept after the PPRX formation.

### Preparation of CD PPRXs with DOX/PEG-LP

**Figure 2:**
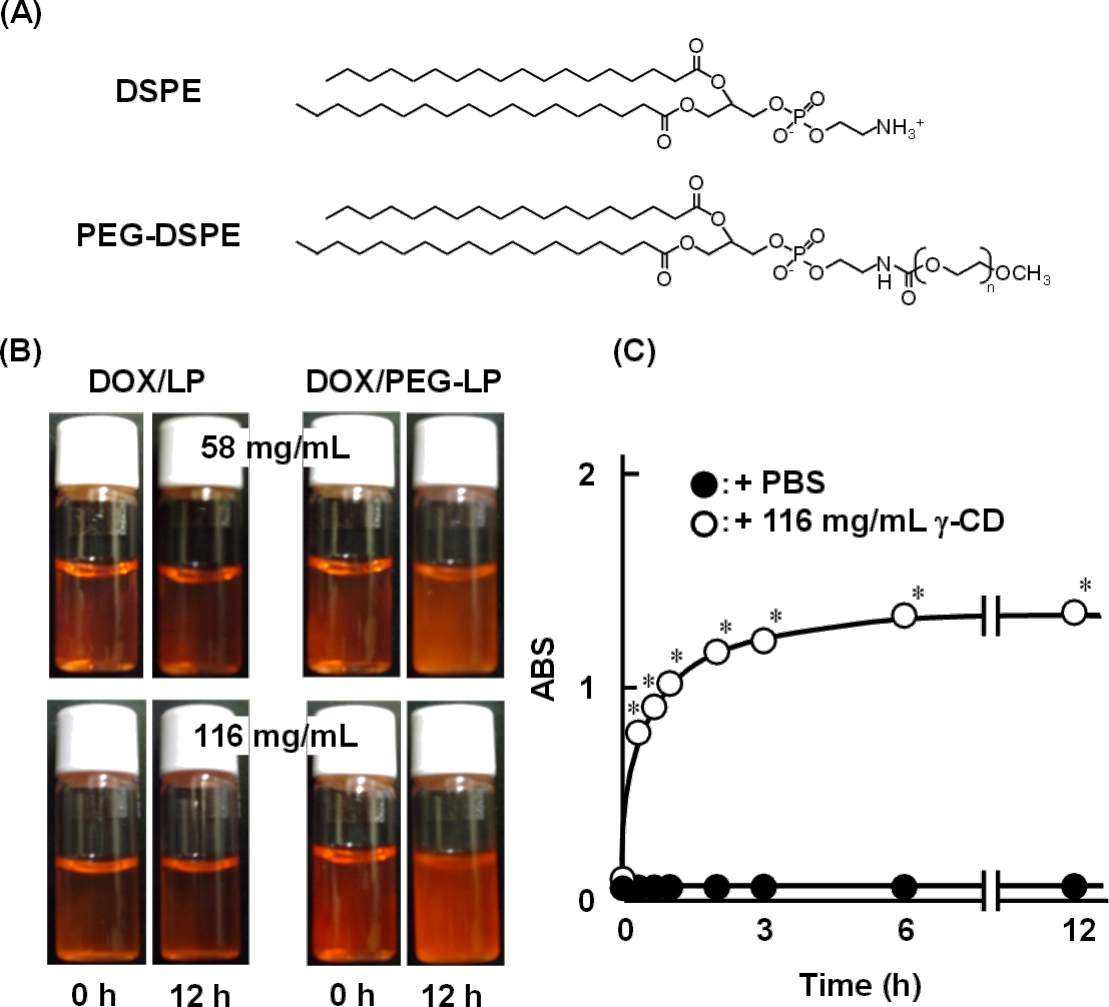
(A) Chemical structures of DSPE and PEG-DSPE, (B) photographs of the γ-CD solutions after adding DOX/LP or DOX/PEG-LP, and (C) turbidity of the DOX/PEG-LP solution in the absence and in the presence of 116 mg/mL γ-CD. Each point represents the mean ± S.E. of 3 experiments. *p < 0.05 versus PBS.

On the basis of the results of Figure 1, we prepared PPRX of DOX/PEG-LP with 58 and 116 mg/mL γ-CD solution. Here, DOX/PEG-LP was prepared with HSPC/cholesterol/PEGylated 1,2-distearoyl-sn-glycero-3-phosphoethanolamine (PEG-DSPE, Figure 2A) (47:47:6, molar ratio). In addition, the particle size, polydispersity index (PDI), ζ-potential and the drug entrapment ratio of DOX/PEG-LP were 132 ± 1.53 nm, 0.07 ± 0.00, 0.51 ± 0.06 and 99.6 ± 0.07%, respectively. When 58 and 116 mg/mL γ-CD solutions were added to DOX/PEG-LP solution, precipitates were provided within 12 h. Meanwhile, no precipitate was observed in the DOX/LP system, which suggests no formation of insoluble precipitate composed of γ-CD and phospholipids (Figure 2B). Moreover, the turbidity of the DOX/PEG-LP solution was increased in the presence of 116 mg/mL γ-CD, but not in the presence of PBS (Figure 2C). These results suggest that γ-CD forms PPRX with DOX/PEG-LP at 58 or 116 mg/mL without a disruption of the membrane integrity of PEG-LP.

Table 1 shows approximate particle sizes, PDI and ζ-potentials of the precipitates obtained by mixing of γ-CD and PEG-LP or DOX/PEG-LP solutions. In both systems, γ-CD formed micro-sized particles with PEG-LP or DOX/PEG-LP, exhibiting almost negligible ζ-potentials, and an obvious difference was not observed. These results suggest that the presence of DOX in PEG-LP scarcely alters the physicochemical properties of the PPRX. Additional studies by means of SEM or TEM may permit a more precise analysis and might confirm whether the LP structure is retained.

**Table 1:** Particle sizes, PDI and ζ-potentials of γ-CD PPRXs with PEG-LP and DOX/PEG-LP.^a^

System	Mean diameter (nm)	PDI	ζ-Potential (mV)

PEG-LP/γ-CD	2543 ± 112	0.22 ± 0.05	−0.50 ± 0.20
DOX/PEG-LP/γ-CD	3097 ± 317	0.09 ± 0.04	0.28 ± 0.29

^a^Each value represents the mean ± S.E. of 3 experiments.

### Structure of CD PPRXs with PEG-LP

**Figure 3:**
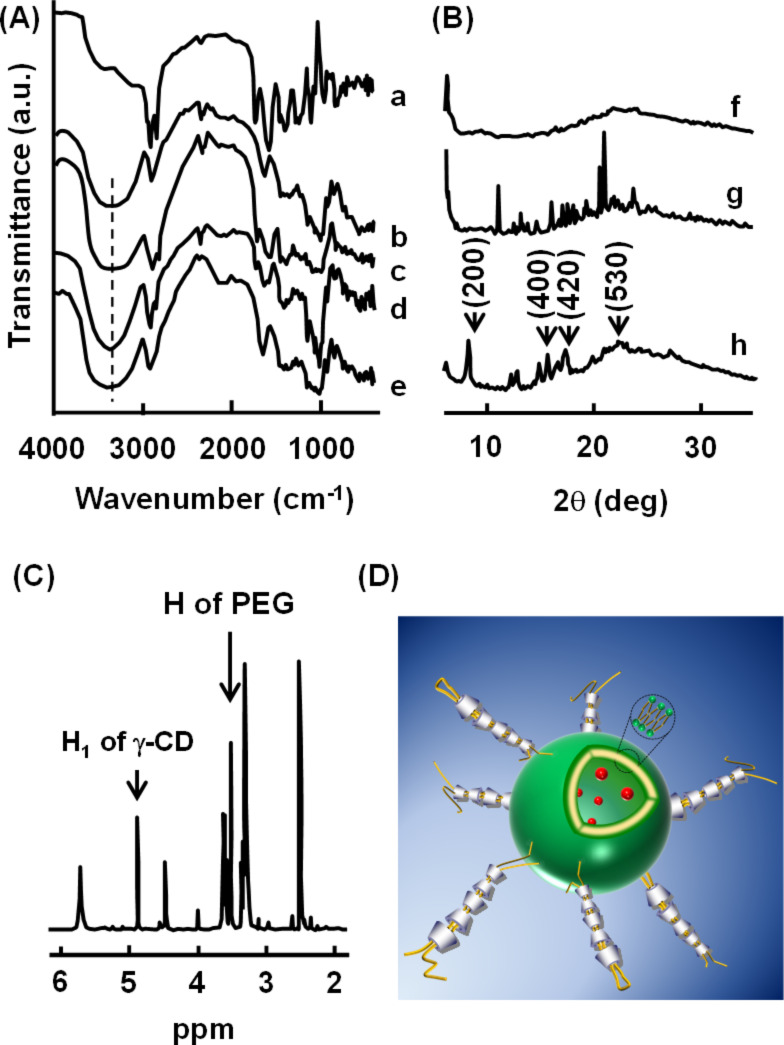
(A) FTIR spectra, (B) powder X-ray diffraction patterns, (C) ^1^H NMR spectrum and (D) proposed partial structure of DOX/PEG-LP/γ-CD PPRX. (a), (f) DOX/PEG-LP, (b) γ-CD alone, (c), (g) DOX/PEG-LP/γ-CD physical mixture, (d), (h) DOX/PEG-LP/γ-CD PPRX, (e) PEG/γ-CD PPRX.

To confirm whether the precipitate obtained in Figure 2 is CD PPRX with DOX/PEG-LP, we first examined its structure by using a FTIR spectrometer. A broad peak observed at 3,354 cm^−1^ of γ-CD in its physical mixture with DOX/PEG-LP was slightly shifted to 3,390 cm^−1^ in the DOX/PEG-LP/γ-CD system (Figure 3A). This shift is probably caused by the hydrogen bonds of the O–H groups of γ-CD derived from PPRX formation [43].

There are three types of crystal packing of CD complexes, namely, the channel type, the cage type and the layer structure [44]. The powder X-ray diffractograms are useful for the confirmation of the PPRXs with CDs, as they provide enough information to distinguish between the herringbone packing of free CDs and the channel packing of inclusion complexes [13]. Therefore, the crystal structure of DOX/PEG-LP/γ-CD was determined by powder X-ray diffraction (Figure 3B). The diffraction peaks were observed at 2θ = 7.43°, 14.16°, 16.65° and 21.87° in the DOX/PEG-LP/γ-CD system. Also, this diffraction pattern was different from that of the physical mixture of DOX/PEG-LP/γ-CD and was almost the same as PEG/γ-CD PPRX used as positive control (Figure 1B, h).

Moreover, the diffraction pattern of DOX/PEG-LP/γ-CD resembled the pattern of tetragonal columnar channels of the linearly aligned γ-CD cavities in the crystalline phase [11,36]. Therefore, the diffraction pattern of the DOX/PEG-LP/γ-CD was indexed on the basis of the two-dimensional tetragonal unit cells with dimensions *a* = *b* = 23.76 Å as shown in Table 2. The *d*-spacing value of the *hkl* (200) reflection was used to calculate the unit cell dimension. The calculated *d*-spacing (*d*_cal_) values were in excellent agreement with those observed (*d*_obs_) suggesting that DOX/PEG-LP/γ-CD forms the tetragonal columnar structure. The results obtained by powder X-ray diffraction patterns suggest that γ-CD includes the PEG chain of DOX/PEG-LP and forms PPRX with tetragonal columnar structure. Additional experiments and analyses are required to determine the tetragonal columnar structure of DOX/PEG-LP/γ-CD PPRX.

**Table 2:** Crystallographic characteristics of γ-CD PPRX with DOX/PEG-LP.

2θ (deg)	(*hkl*)	*d*_obs_ (Å)	*d*_cal_^a^ (Å)

7.43	(200)	11.88	11.88
14.16	(400)	5.93	5.94
16.65	(420)	5.32	5.31
21.87	(530)	4.06	4.07

^a^Calculated assuming a tetragonal unit cell with *a* = *b* = 23.76 Å.

Next, to confirm the stoichiometry of the γ-CD PPRX with DOX/PEG-LP, a ^1^H NMR spectrum was measured (Figure 3C). Approximately 12 mol of γ-CD were involved in the PPRX formation with one PEG chain in the DOX/PEG-LP, which indicates that four ethylene glycol repeating units are included in one γ-CD cavity [9]. In the case of α-CD, two ethylene glycol repeating units of PEG are included in one α-CD cavity resulting in the formation of PPRX with one PEG chain [6–7]. Therefore, γ-CD likely forms PPRX with two PEG chains and/or one bending PEG chain in DOX/PEG-LP.

Figure 3D shows the proposed schematic structure of γ-CD PPRX with DOX/PEG-LP taking into consideration the results of Figure 3. γ-CD includes two PEG chains and/or one bending PEG chain of DOX/PEG-LP and forms PPRX. The PPRX moieties pack with a tetragonal columnar structure.

### Release profile of DOX or DOX/PEG-LP from the CD PPRX

**Figure 4:**
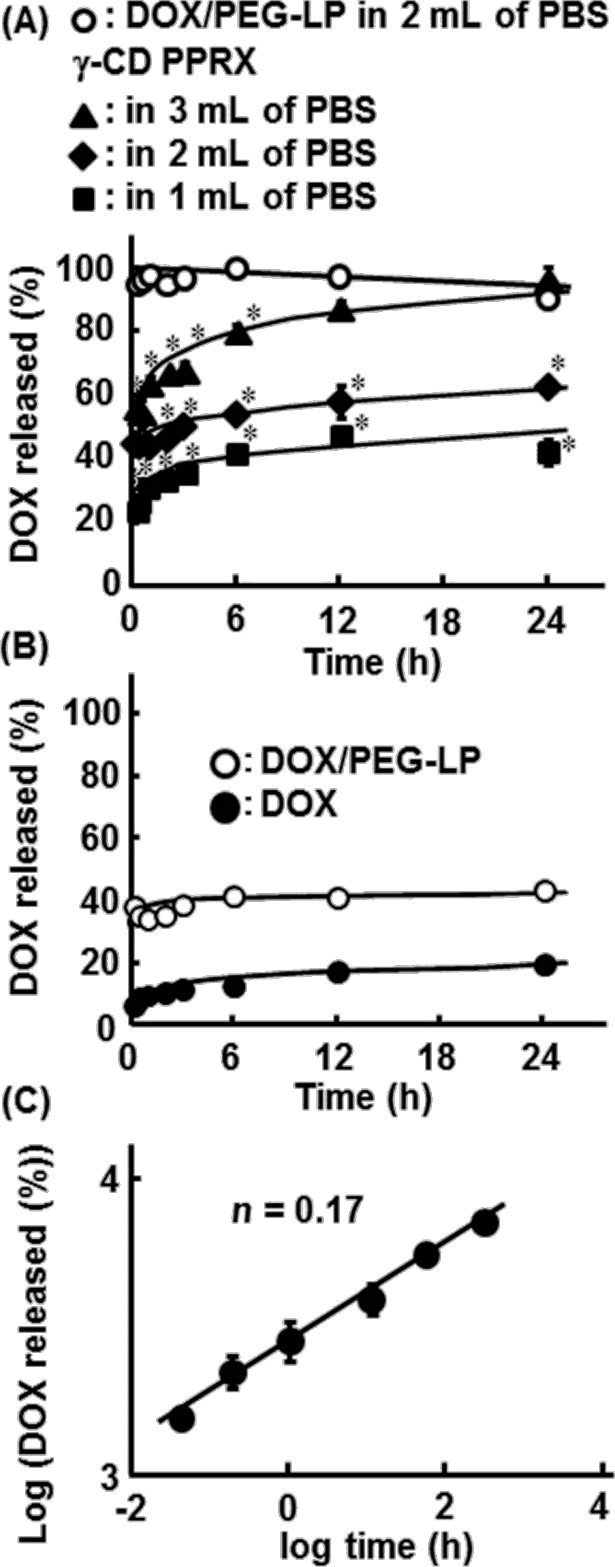
(A) In vitro release profiles of the total DOX from γ-CD PPRX in various volumes of PBS, (B) release profiles of each naked DOX and DOX/PEG-LP in 2 mL of PBS, and (C) Korsmeyer–Peppas release profile of the total DOX from γ-CD PPRX. The amount of DOX was 0.2 mg to 1 mg of lipid. Each point represents the mean ± S.E. of 3 experiments. *p < 0.05 versus DOX/PEG-LP in 2 mL of PBS.

To evaluate the applicability of γ-CD PPRX with DOX/PEG-LP as a sustained release carrier for DOX, the release profile of DOX or DOX/PEG-LP from the CD PPRX was examined. Firstly, the total amount of DOX including naked DOX and encapsulated DOX in PEG-LP was quantitatively detected by fluorescence spectrometry. As shown in Figure 4A DOX/PEG-LP was rapidly dissolved in PBS. On the other hand, the release of naked DOX or DOX/PEG-LP from γ-CD PPRX was prolonged and was accelerated by the increase of the volume of the dissolution medium (the rate: 3 mL > 2 mL > 1 mL). Next, naked DOX and encapsulated DOX in PEG-LP were separately detected, and their release profiles were investigated (Figure 4B). Approximately 10–20% and 30–40% of DOX were released as naked DOX and encapsulated DOX in PEG-LP, respectively, in the 2 mL of dissolution medium system (Figure 4B). These results suggest that γ-CD PPRX with DOX/PEG-LP shows the sustained release profile of DOX.

To investigate the release mechanism of naked DOX or DOX/PEG-LP from γ-CD PPRX, the release kinetic profiles were applied to kinetic models such as zero order, first order, Higuchi and Hixson–Crowell equations [45]. The drug-release data obtained within 12 h in 1 mL of dissolution medium system were used. The release rate constants were calculated from the slope of the appropriate plots, and the correlation coefficient (*r*) was also calculated (Table 3). The release kinetics of naked DOX or DOX/PEG-LP from γ-CD PPRX was well-fitted to the Higuchi’s equation with a high *r* value of 0.90, indicating that the release shows the matrix type release profile.

**Table 3:** Release constants and *r* values calculated from zero order, first order, Higuchi and Hixson–Crowell equations.

Release model	Parameter	Value^a^

Zero order	*k*_0_ (%/h) *r*	1.72 ± 0.06 0.81 ± 0.03
First order	*k*_1_ (h^−1^) *r*	0.03 ± 0.00 0.84 ± 0.03
Higuchi	*k*_H_ (%/h^1/2^) *r*	7.31 ± 0.26 0.90 ± 0.01
Hixson–Crowell	*k*_s_ (%^1/3^/h) *r*	0.03 ± 0.01 0.83 ± 0.03

^a^Each value represents the mean ± S.E. of 3 experiments.

In the matrix type release profile, the bases are either hydrophilic or hydrophobic. If a base is hydrophilic, the erosion and the dissolution of a base are observed among the drug release, and the Korsmeyer–Peppas model is often used. Therefore, we analyzed the release profile of naked DOX or DOX/PEG-LP from γ-CD PPRX with the Korsmeyer–Peppas model which was developed to specifically model the release of a drug from a polymeric matrix. As shown in Figure 4C, naked DOX or DOX/PEG-LP released from γ-CyD PPRX showed a fair linearity, and the exponent value (*n*) was 0.17. In this model, the *n* value characterizes the release mechanism of a drug. For instance, *n* < 0.5, *n* = 0.5 and 0.5 < *n* ≤ 1 correspond to a quasi-Fickian, Fickian and non-Fickian diffusion mechanism, respectively [46]. Therefore, the release of naked DOX or DOX/PEG-LP released from γ-CD PPRX is probably in accordance with a quasi-Fickian diffusion mechanism resulting from a coupling of the diffusion and erosion mechanism, and is controlled by more than one process [46]. Taken together, the proposed mechanism for the release of naked DOX or DOX/PEG-LP from γ-CD PPRX may be based on the dethreading of γ-CD from the PEG chain through the dilution, resulting in the erosion of γ-CD PPRX and diffusion of the drug.

## Conclusion

In the present study, we first investigated the formation of γ-CD PPRX with DOX/PEG-LP without disruption of the LP membrane integrity. γ-CD formed PPRX characterized by a tetragonal columnar structure with DOX/PEG-LP through the inclusion complexation with two PEG chains and/or one bending PEG chain of DOX/PEG-LP. Moreover, the resulting PPRX showed a sustained release profile of DOX and/or DOX/PEG-LP. To clarify detailed structures of γ-CD PPRX with DOX/PEG-LP and its utility as a sustained release drug carrier, further studies are required. Especially, differences in the structures of γ-CD PPRX with DOX/PEG-LP could exert influence on the release rate of DOX/PEG-LP. It may be worthwhile to investigate the controlled release system of DOX/PEG-LP by means of the formation of PPRXs with various structures. Nevertheless, our findings provide useful information to design a novel sustained release system for PEG-LP products.

## Experimental

### Materials

α- and γ-CDs were donated by Nihon Shokuhin Kako (Tokyo, Japan). DOX hydrochloride was purchased from Wako Pure Chemical Industries (Osaka, Japan). HSPC (COATSOME^TM^ NC-21E), DSPE (COATSOME^TM^ ME-8080) and PEG-DSPE (SUNBRIGHT^TM^ DSPE-020-CN, molecular weight of PEG = 2,000) were purchased from NOF corporation (Tokyo, Japan). Other chemicals and solvents were of analytical reagent grade.

### Interaction of CDs with HSPC

HSPC (50 mg) was dissolved in 3 mL of chloroform. After evaporation and drying under reduced pressure overnight, 3 mL of water were added. The resulting suspension (100 μL) was added to 900 μL of α-CD (145 mg/mL) or γ-CD (232 mg/mL) aqueous solution, and the suspension was kept at 4 °C for 12 h. After centrifugation (12,000 rpm, 10 min), the supernatant was removed. The resulting precipitate was dried under reduced pressure, and then the powder X-ray diffraction was measured.

### Powder X-ray diffraction

Powder X-ray diffraction patterns were measured by a Rigaku Ultima IV X-ray diffractometer (Tokyo, Japan) with a Ni filtered Cu Kα radiation, a voltage of 40 kV, a current of 40 mA, a scanning speed of 5°/min, a time constant of 2 s, and a scan range of 2θ = 5–35°.

### Effects of CDs on the entrapment ratio of DOX into LP or PEG-LP

DOX/PEG-LP was prepared with HSPC/cholesterol/PEG-DSPE (47:47:6, molar ratio) according to the method previously reported by Arima et al. [47]. The DOX/PEG-LP solution (20 μL) was added to 500 μL of α-CD (36.3, 72.5 and 145 mg/mL) or γ-CD (58.0, 116 and 232 mg/mL) PBS solution, and the suspension was kept at 4 °C for 12 h. After 20-fold dilution with PBS and ultracentrifugation (50,000 rpm, 60 min), the fluorescence intensity of the supernatant was measured by a F-4500 fluorescence spectrometer (Hitachi, Tokyo, Japan) at λ_em_ = 554 nm (λ_ex_ = 470 nm).

### Preparation of CD PPRX with DOX/PEG-LP

The DOX/PEG-LP solution (40 μL) was added to 1000 μL of γ-CD (58.0 or 116 mg/mL) PBS solution, and the suspension was kept at 4 °C for 12 h. The turbidity of the resulting suspension was measured with a JASCO V-630 UV–visible spectrophotometer (Tokyo, Japan) at 800 nm. To obtain the solid sample of the PPRX, the supernatant was removed after the centrifugation (12,000 rpm, 10 min). The resulting precipitate was dried under reduced pressure.

### Particle size, PDI and ζ-potential of CD PPRX with DOX/PEG-LP

The particle size, PDI and ζ-potential of the suspension of γ-CD PPRX with DOX/PEG-LP were determined by dynamic light scattering by using a Zetasizer Nano (Malvern Instruments, Worcestershire, UK). The dynamic light scattering was analyzed by the general purpose mode. The measurements were carried out at least in triplicates.

### FTIR

The sample preparation was performed by using the KBr method. The FTIR spectrum of γ-CD PPRX with DOX/PEG-LP was recorded on a JIR-6500W FTIR spectrometer (JEOL, Tokyo, Japan) in the range between 4,000 and 400 cm^−1^ with a resolution of 4 cm^−1^ and 16 scans.

### ^1^H NMR

^1^H NMR spectrum was taken at 25 °C on a JEOL α-500 FT-NMR (Tokyo, Japan) operating at 500 MHz by using a 5 mm sample tube. Deuterated DMSO (DMSO-*d*_6_) was used as a solvent. The stoichiometry of the γ-CD PPRX with DOX/PEG-LP was determined by measuring peak areas of the anomeric proton of CDs and the ethylene protons of the PEG-LP.

### In vitro release study

The various volumes (1.0, 2.0 or 3.0 mL) of PBS (pH 7.4) were added to the suspension including γ-CD PPRX with DOX/PEG-LP at 37 °C and stirred at 100 rpm. At appropriate intervals, 100 μL of the dissolution medium were withdrawn, centrifuged at 5,000 rpm for 5 min, and 20-fold diluted with PBS. After the addition of Triton^TM^ X-100 (10 μL), the samples were analyzed by a F-4500 fluorescence spectrometer (Hitachi, Tokyo, Japan) at λ_em_ = 590 nm (λ_ex_ = 470 nm).

The release kinetics of DOX or DOX/PEG-LP from the γ-CD PPRX was evaluated according to zero order kinetics, first order kinetics, Higuchi’s model, Hixson–Crowell model and Korsmeyer–Peppas’s model.

Zero order kinetics

[1]
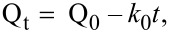


where Q_t_ is the amount of drug remaining in solid state at time *t*, Q_0_ is the initial amount of drug in the γ-CD PPRX and *k*_0_ is the zero order release rate constant.

First order kinetics

[2]
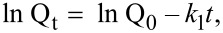


where Q_t_ is the amount of drug remaining in solid state at time *t*, Q_0_ is the initial amount of drug in the γ-CD PPRX and *k*_1_ is the first order release rate constant.

Higuchi’s model

[3]
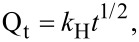


where Q_t_ is the amount of drug released in time *t* and *k*_H_ is the Higuchi’s (release) rate constant.

Hixson–Crowell model

[4]
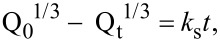


where Q_0_ is the initial amount of drug in the γ-CD PPRX, Q_t_ is the amount of drug remaining in solid state at time *t* and *k*_S_ is the Hixson–Crowell (release) rate constant.

Korsmeyer–Peppas’s model

[5]
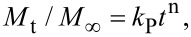


where *M*_t_/*M*_∞_ is fraction of drug release at time *t*, *k*_P_ is the release rate constant, and n is the release exponent.

### Data analysis

Data were given as the mean ± S.E. The statistical significance of mean coefficients for the studies was performed by analysis of variance followed by Scheffe's test. *p*-Values for significance were set at 0.05.
